# Disseminated lung cancer presenting as a rectal mass

**DOI:** 10.3402/ecrj.v3.31726

**Published:** 2016-09-27

**Authors:** Mia M. Noergaard, Inger M. H. Stamp, Uffe Bodtger

**Affiliations:** 1 Department of Pulmonology, Naestved Hospital, Naestved, Denmark; 2 Department of Pathology, Naestved Sygehus, Naestved, Denmark; 3 Institute of Regional Health Research, University of Southern Denmark, Odense, Denmark

**Keywords:** non-small cell lung cancer, endoscopy, diagnosis, rectum, histology

## Abstract

Primary lung cancer is the leading cause of cancer-related deaths globally, and approximately 50% had metastatic disease at the time of diagnosis. A rectal mass and unintended weight loss are common manifestations of rectal cancer. Our case presented with a rectal mass, but workup revealed a metastatic lesion from lung cancer. Lung cancer metastases to the lower gastrointestinal tract imply reduced survival compared with the already poor mean survival of stage IV lung cancer. Despite relevant therapy, the patient died 5 months after referral.

Primary lung cancer is the leading cause of cancer-related deaths globally. Metastatic disease at diagnosis is very common, typically involving liver, brain, adrenals, and bones. Symptomatic intestinal/colonic metastases affect 0.2–0.5% (5% in autopsy studies) but imply a median survival of 3 months compared with 8 months in overall stage IV non-small cell lung cancer (1, 2). This case presented with a rectal mass, a common clinical manifestation of another common malignancy, colorectal cancer. The case illustrates the unpredictability of malignant spread, and the occasional mismatch between clinical presentation and tissue sampling results.

## Case presentation

A 70-year-old male never-smoker experienced an 8-kg weight loss during 6 months, alternating diarrhoea/obstipation without bloody stools, slight coughing, and dyspnoea. He had a history of hypertension for many years, uncomplicated and treated with losartan/thiazide 50/12.5 mg and amlodipine 10 mg once daily. Nine years earlier, he was diagnosed with a transitiocellular, papillary bladder tumour, grade III with invasion; underwent successful endoscopic resection without need for oncological treatment; and was followed with annual cystoscopy. Three years later, he was surgically treated for a relapse of transitiocellular, papillary bladder tumour (grade I) and was followed for an additional 5 years without signs of recurrent disease at annual cystoscopy.

He was a retired industrial worker but with no known occupational exposure to asbestosis, silica, or dust. He was living with his spouse and had no hobbies resulting in exposure to hazardous fumes or dusts.

## Diagnostic workup

**Fig. 1:**
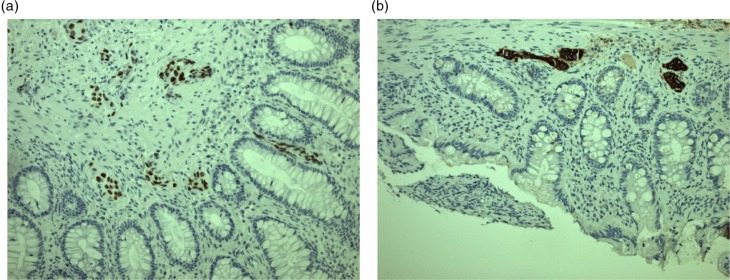
Immunohistochemical staining of histological biopsy from rectal mass: an adenocarcinoma positive for TTF-1 (A) and CK7 (B).

He was referred to an outpatient colonoscopy (17 months after he ended urological control visits), revealing an obstructing lower rectal mass. Only a paediatric coloscope could pass the lesion, and biopsies revealed adenocarcinoma, immunohistochemical (IHC) analyses positive for CK7 and TTF-1 (Fig. 1), and negative for GATA, CK20, CDX2, PLAP, CD117, PSA, and S100P. PCR showed wild-type EGFR status. Direct histological comparison showed no resemblance with his urological cancer.

**Fig. 2:**
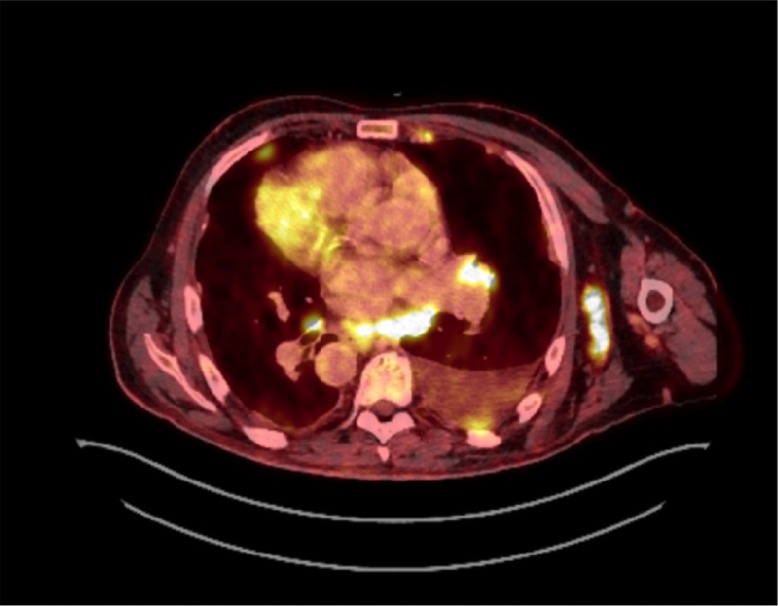
Chest PET-CT scan showing no sign of a primary lung lesion but multiple enlarged lymph nodes, a left-sided compression atelectasis, and ipsilateral pleural effusion.

**Fig. 3:**
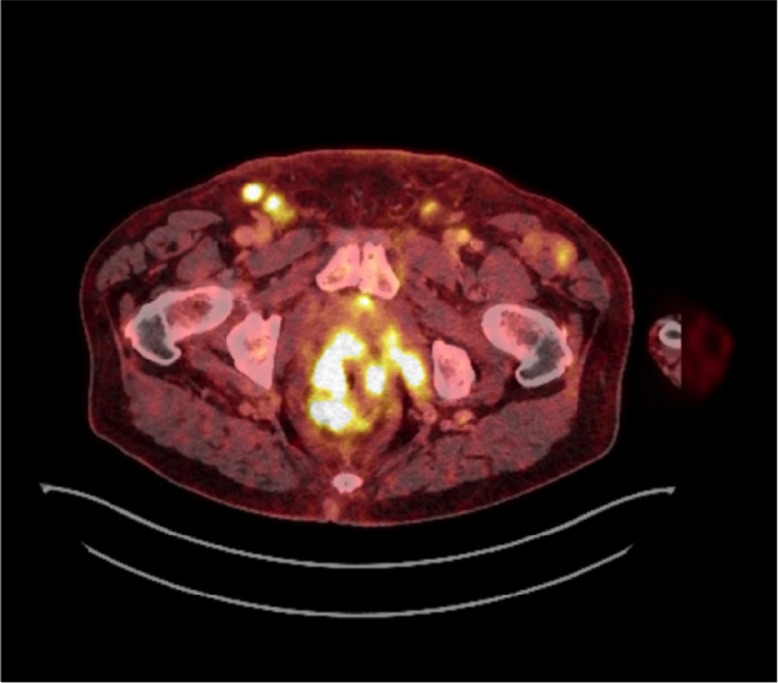
Abdominal PET-CT scan showing a rectal mass, moderate quantities of ascites, and enlarged lymph nodes in pelvis and groin.

Thoracoabdominal contrast-enhanced PET-CT scan showed multiple, PET-positive, enlarged mediastinal and retroperitoneal lymph nodes, a right-sided pleural effusion and associated ipsilateral compression lower lobe atelectasis, ascites, and a tumour located in the rectum with invasion to levator muscles and vesicula seminalis (Figs. 2 and 3). Cytological sampling from mediastinal lymph nodes and pleural exudate showed identical adenocarcinoma including IHC profile cells with a high proliferation index assessed by Ki-67. A bronchoscopy showed no visual tumour.

At a multidisciplinary team conference, it was decided that the final diagnosis was primary lung cancer, stage TxN3M1b with local metastases to mediastinal lymph nodes, pleura, rectum, and peritoneum. The IHC results were considered to rule out primary colorectal cancer or vesical cancer with thoracic dissemination.

He started palliative carboplatin/vinorelbine and avastin (43 days after referral to gastroenterologist) as 4 series with 3 weeks intervals. Three-month PET-CT showed a mixed response with an overall sign of progression, and second-line chemotherapy was planned. Before onset, he was admitted with acute dyspnoea and died 2 days later.

## Discussion

The current case describes a rare site of primary lung cancer metastasis with fatal outcome 153 days after referral. Despite an unusual presentation, neither diagnosis nor therapy onset was delayed.

One can question the validity of the diagnosis as the patient was a never-smoker without evident lung lesions. However, as three key IHC markers for colorectal cancer were negative, positive CK7 and TTF-1, and no upper G-I CT/PET lesions, neither a primary upper or lower G-I cancer seems likely (3). We found three published case reports on rectal metastases from lung cancer: two were late metastases in earlier surgically resected patients (4, 5), and a recently published case on metastatic, poorly differentiated squamous cell lung carcinoma with multiple metastases to liver, skin, bone, and muscle (6).

It is unresolved if intestinal spread reflects a more aggressive or more advanced disease. G-I metastasis may occur through haematogenic spread via the mesenteric inferior artery supplying descending colon, sigmoideum and superior part of rectum (7, 8). Yet, cancer metastasis is complex, involving coordination of expression/suppression of numerous genes, and is not fully understood. IL-8 overexpression may contribute to a more aggressive phenotype yet our case was negative for CK20, the IL-8 receptor (9).

Improved survival after intestinal single-metastasis resection has been demonstrated in carefully selected patients, but for most patients with intestinal metastases, palliation remains the only available therapy (10).

An improved understanding of the pathobiological mechanisms involved in cancer metastases is needed to elucidate targets for improved therapy.
